# Selected annotated instance segmentation sub-volumes from a large scale CT data-set of a historic aircraft

**DOI:** 10.1038/s41597-024-03347-4

**Published:** 2024-06-24

**Authors:** Roland Gruber, Nils Reims, Andreas Hempfer, Stefan Gerth, Michael Böhnel, Theobald Fuchs, Michael Salamon, Thomas Wittenberg

**Affiliations:** 1 Fraunhofer IIS, Fraunhofer Institute for Integrated Circuits IIS, Division Development Center X-Ray Technology, Fürth, Germany; 2 Friedrich-Alexander-Universität Erlangen-Nürnberg, Chair for Visual Computing, Erlangen, Germany; 3 Deutsches Museum, München, Germany

**Keywords:** X-rays, Computer science, Scientific data

## Abstract

The Me 163 was a Second World War fighter airplane and is currently displayed in the *Deutsches Museum* in Munich, Germany. A complete computed tomography (CT) scan was obtained using a large scale industrial CT scanner to gain insights into its history, design, and state of preservation. The CT data enables visual examination of the airplane’s structural details across multiple scales, from the entire fuselage to individual sprockets and rivets. However, further processing requires instance segmentation of the CT data-set. Currently, there are no adequate computer-assisted tools for automated or semi-automated segmentation of such large scale CT airplane data. As a first step, an interactive data annotation process has been established. So far, seven 512 × 512 × 512 voxel sub-volumes of the Me 163 airplane have been annotated, which can potentially be used for various applications in digital heritage, non-destructive testing, or machine learning. This work describes the data acquisition process, outlines the interactive segmentation and post-processing, and discusses the challenges associated with interpreting and handling the annotated data.

## Background & Summary

**Fig. 1:**
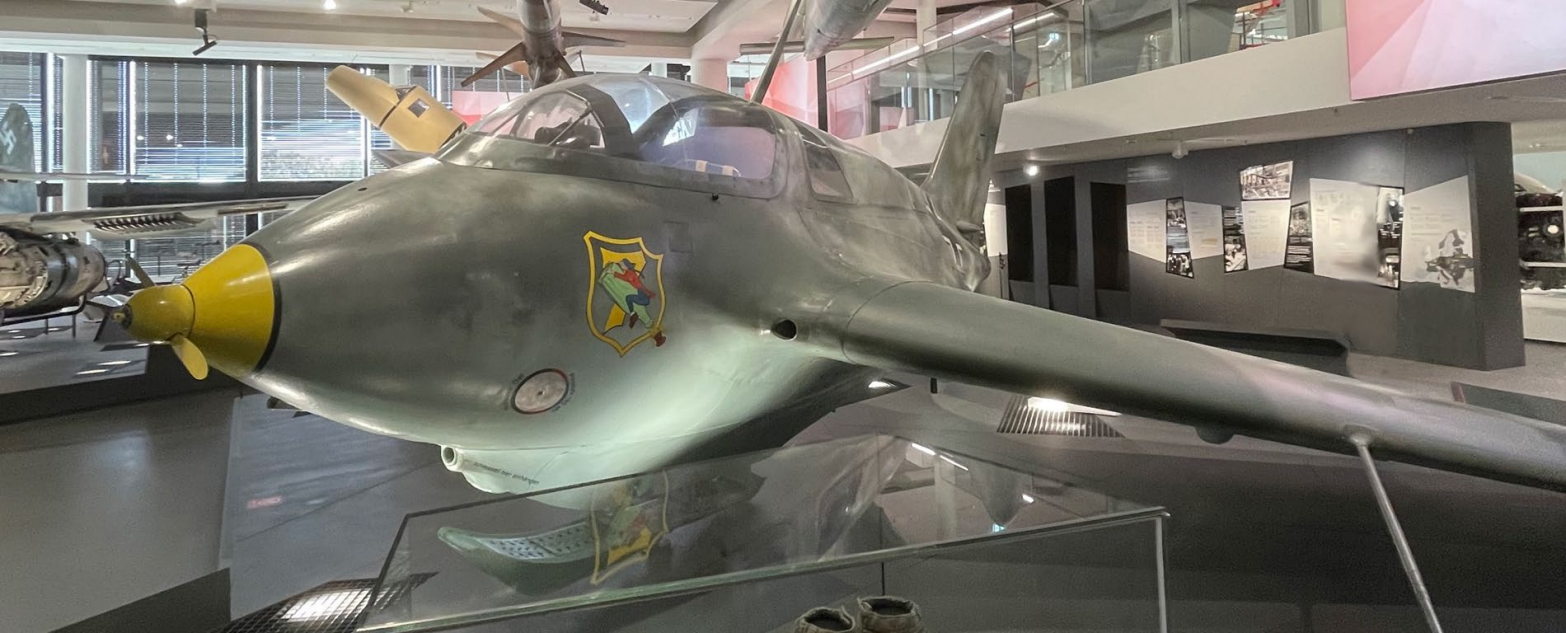
Image of the Messerschmitt Me 163 in the historic aircraft exhibition of the *Deutsches Museum*.

The Messerschmitt Me 163, see Fig. 1, was a German fighter airplane with a rocket engine during the Second World War, and was part of the secret developments of the German air force^1^. With its unique rocket engine, it was the first piloted aircraft to reach a maximum speed of about 1000 km h^−1^. Of the 350 Me 163 s built between 1941 and 1945, only ten examples survive in museums, one of which is displayed in the in the historic aircraft exhibition of the *Deutsches Museum* in Munich, Germany.

**Fig. 2:**
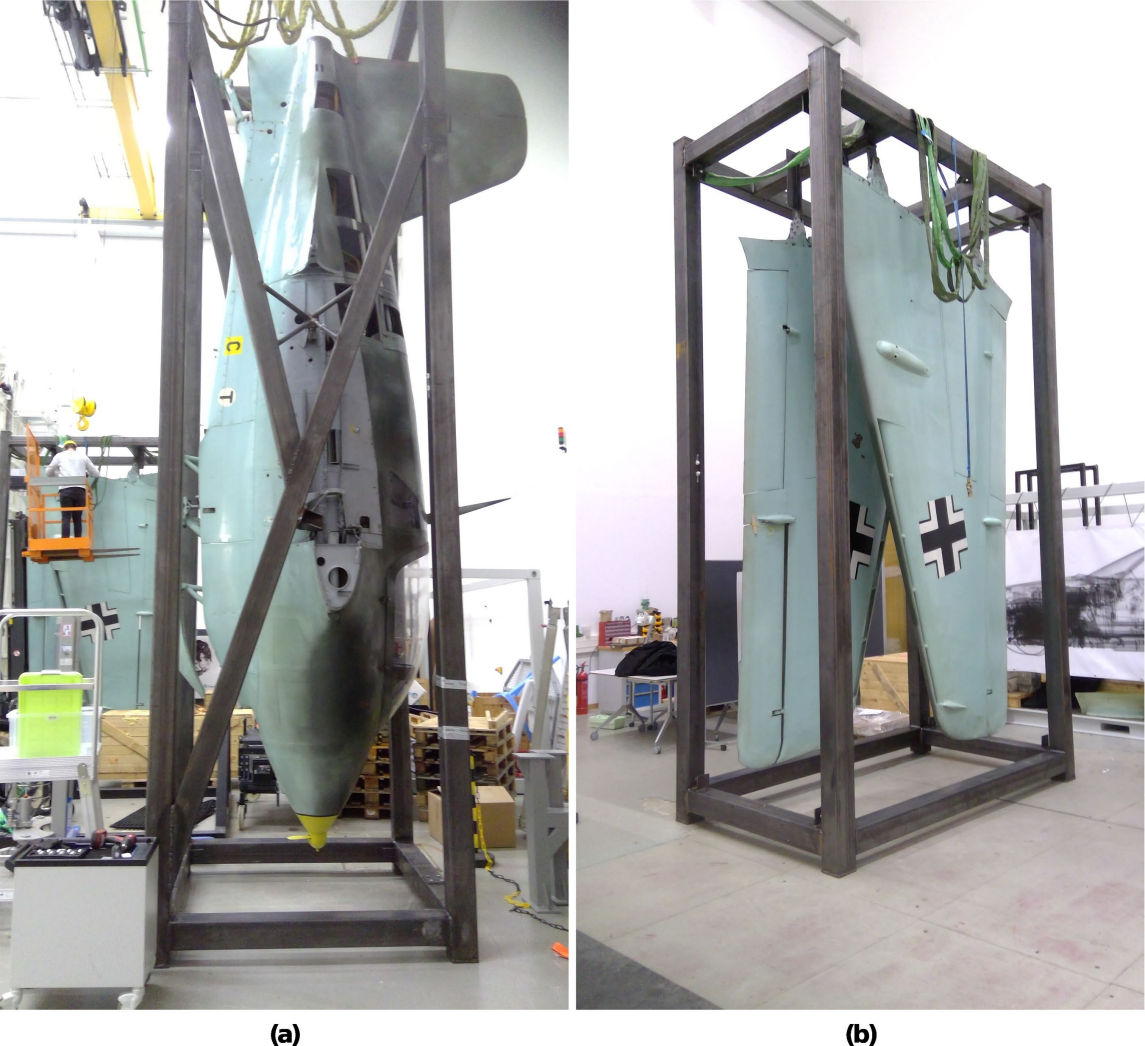
Fuselage (**a**) and wings (**b**) of the Me 163 airplane inside the mounting brackets for the CT scan.

To gain new insights into the history, design and state of preservation of this unique and historical airplane, a complete CT scan (see Fig. 2) was obtained. The CT scan served two main purposes. Firstly, it aimed to identify this particular airplane, as the airplane lacked any production number or other unique identifiers accessible without disassembling the whole airplane. Secondly, the CT scan should provide insight into history, design and state of preservation of this unique and historical airplane. This includes understanding the production components used and any modifications made throughout the airplane’s lifespan starting from it’s time in Germany during the Second World War until it’s donation to the *Deutsches Museum* in 1964 by the British Royal Air Force (RAF), which possessed the airplane after the war.

The CT data is being complemented with archival research, and this ongoing work is expected to contribute further to understanding the airplane’s history. Additionally, the CT data-set is used to enhance the visitors experience in the aviation exhibition of the *Deutsches Museum*. Active thermography inspections of this plane have been carried out^2^ and provide additional background information on the plane.

Besides viewing and examining the large scale CT data in detail using adequate interactive volume reader and viewer software^3^ also, the airplane’s many individual components are of interest, such as the screws, wheels, sprockets, rivets, and much more.

To obtain more information about these parts, their distribution within the airplane and their spatial and functional relationships to each other ideally an automated, semi-automatic, or purely manual instance segmentation or partitioning of all components and objects of interest into disjoint parts from the CT data is necessary.

To this end, different automated CT volume-segmentation methods of different complexity could be applied to obtain a set of segmented airplane parts. Nevertheless, all automatic as well as semi-automatic volumetric image segmentation methods usually depend strongly on the availability of sufficient and adequate labelled reference data needed for the parameter optimisation or parameter training, as well as the sufficient evaluation of the developed delineation approaches. As the Me 163 airplane is an example of a unique object with partially very exceptional and matchless components, of which only one CT-scan exits (also known as *lot-one-problem*), adequate automatic or semi-automatic segmentation methods are currently not available, enabling and supporting the delineation of the airplane’s different components.

However, if–as a first step–some adequate labelled reference data from such a large scale CT airplane scan would be available, the development of new segmentation methods, either based on traditional image processing methods or alternatively using novel deep-learning approaches (e.g. employing deep convolutional neural networks (DCNNs)^4–6^, could be developed and evaluated more efficiently. Especially as the performance of such DCNNs on vision tasks tends to increase logarithmically based on the volume of training data^7^. Thus, in order to optimize an automation segmentation scheme, a large set of well-curated ground truth data sets is of most importance^8,9^.

Hence, within this contribution, we will provide historical background to the Me 163 and its current stay in the *Deutsches Museum* (Section Me 163), describe the data acquisition process using a large scale CT scanner (Section Data Acquisition), outline the interactive labelling and annotation process of some distinct sub-volumes of the airplane (Section Data Annotation), and discuss various challenges with respect to interpreting and handling the annotated and labelled data (Section Challenges). Furthermore, we introduce a matrix-based metric to compare two (manually or automatic) labelled segmentations (Section Technical Validation) which can handle erroneous split or merged segments as well as voxel overlap of the segments.

Seven of the sub-volumes together with their manually obtained annotations are available^10^ to be used in the future by researchers in the fields of digital heritage, non-destructive testing, machine vision and/or artificial intelligence to visualize and interact with as well as to develop, train, optimise and evaluate novel volumetric instance segmentation approaches.

## Methods

### The Me 163

The Messerschmitt Me 163^1,11,12^ in the historic aircraft collection and exhibition of the *Deutsches Museum* (see Fig. 1) is still a mysterious plane. The RAF gifted it to the museum in 1964, but since the ID plate in the nose is empty, not much is known about its operational history in the Second World War or its second life in Great Britain. After it was captured in 1945, the plane was modified for flight-testing by the RAF. When an accident with another Me 163 nearly killed a test pilot, the Me 163 s were kept only as technological curiosities. Some were scrapped after, some found their way into museums around the globe.

The British had realized that this alleged Nazi “wonder weapon” was more of a danger for its pilots rather than allied planes. Developed from innovative tailless gliders by Alexander Lippisch and fitted with a Walter HWK 109–509 rocket engine with 14.7 kN of thrust in 1941, the Me 163 reached exceptional speeds and climb rates. The small and light airframe with its thick, swept wings reached Mach 0.84 and could climb up to 81 m s^−1^. These achievements, however, came at a very high price: With no space for a retractable landing gear, the wheels were jettisoned after takeoff, often bouncing back and damaging the plane. The rocket fuel was depleted in just seven minutes, leaving very little time to reach the enemy. The armament was weak and unsuited for the purpose of the Me 163, that is intercepting heavy allied bombers. When gliding back to base, pilots could evade attacking fighters thanks to the good maneuverability of the Me 163, only to be sitting ducks after they came to a halt on the landing skid. What really made the plane an unacceptable hazard for pilots and ground crew was its highly flammable rocket fuel: “C-Stoff” and “T-Stoff” (the latter 80% hydrogen peroxide) exploded on contact and fumes could dissolve any organic matter. Fatal accidents at take-off or landing were common.

The Me 163, as well as the so-called weapons “V1” and “V2”, embodies a widespread belief in innovative technology as a miraculous savior from vastly superior allied air power. Forced laborers, willingly exploited by the German industry by the hundreds of thousands, had to build many parts of the plane in murderous conditions. In the end, the approx. 350 Me 163 s produced in total, shot down only nine heavy allied bombers between 1943 and 1945. In telling us about the hubris of its engineers as well as cultural aspects of technology, the Me 163 is a highly sought-after study object.

### Data acquisition

**Fig. 3:**
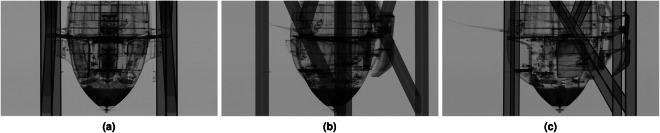
Three X-ray projections of the front part of the airplane fuselage which were captured at different rotation angles: 0° (see **a**), 45° (see **b**), and 90° (see **c**). The vertical dark structures on the outer area of the images represent the mounting brackets for the CT scan. In the centre of the images, the lower part shows the propeller-like generator, and in the middle section, the pilot’s seat along with the dashboard is visible.

The data-set of the historic Me 163 airplane was acquired at the Fraunhofer IIS development center for X-ray technologies EZRT in Fürth, Germany with a unique type of large scale high energy industrial CT system called XXL-CT. To the best of our knowledge, this is the largest CT system available world wide for civil purposes and open to industrial and public applications. This system is capable of handling objects of several meters in length and diameter by combining a linear accelerator, a large manipulation stage, and a line detector array^13^. In first order, the size of the object’s diameter is limited by the 4-meter-width of the horizontally aligned detector array. Therefore, the system can handle huge objects complete such as cars or sea freight containers. Figure 3 displays a series of X-ray projections of the airplane’s fuselage front, each taken at distinct rotation angles to reveal various internal features.To cover the complete airplane, four subsequent CT-scans were performed, two for the fuselage (see Fig. 2a) and two for the disassembled wings (see Fig. 2b).

**Fig. 4:**
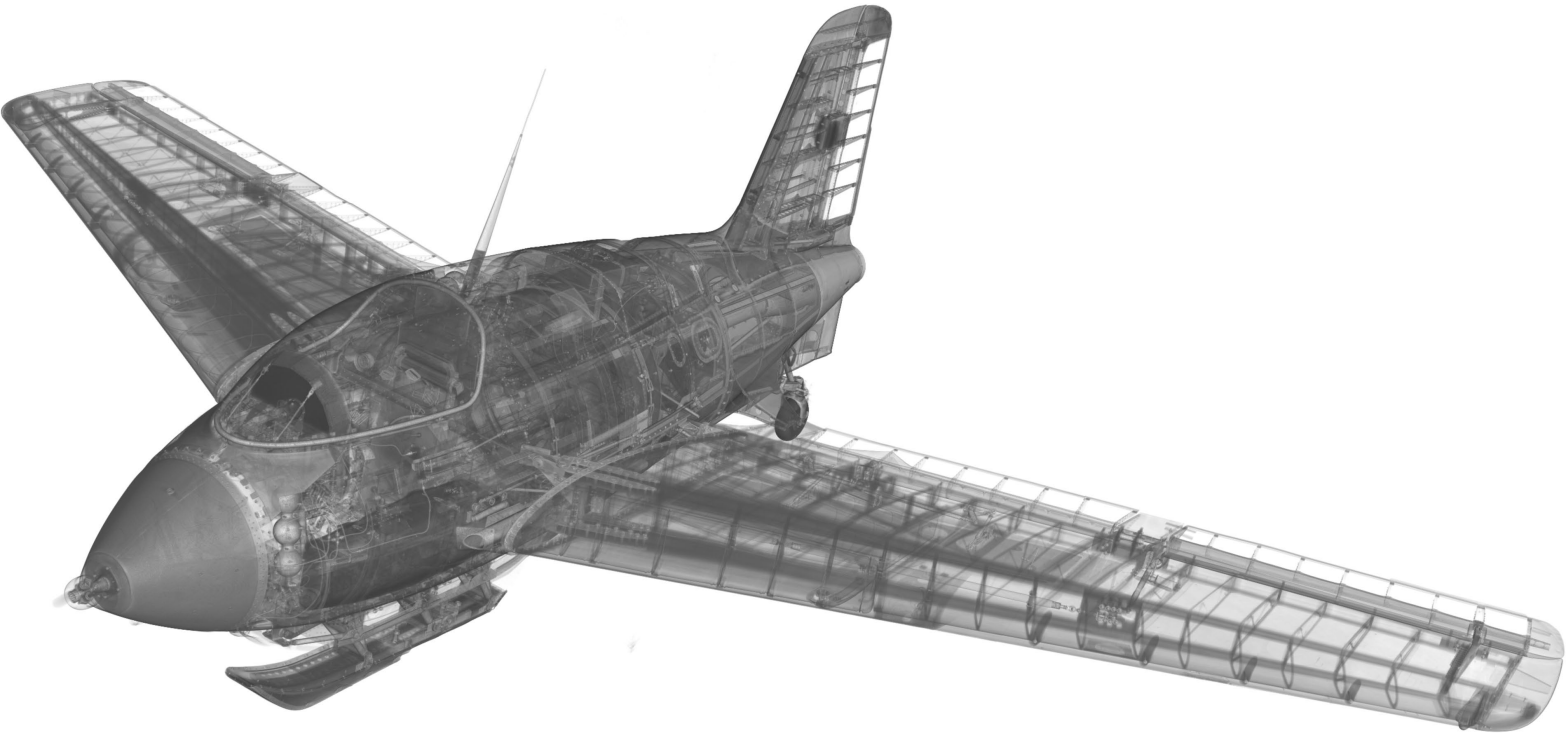
Rendering of the reconstruction of the whole airplane. The fuselage and wings are virtually integrated to form the complete airplane.

Afterwards, the two sub-data-sets for the fuselage and the two sub-data-sets for the wings were manually merged into one data-set for the fuselage and one data-set for the wings. In the further course we focus on the data-set of the airplane fuselage, from which all annotated sub-volumes originate. To provide an overall impression, a rendering of the entire airplane is shown in Fig. 4.

The merging process was carried out manually by carefully aligning the rear part of the fuselage to the front part of the fuselage. To help with this process the CT scan was parameterized in such way that an overlap region is present in both reconstructed data-sets. Fitting high contrast details in the overlap region like metal sheets of different orientations in X,Y and Z onto each other was sufficiently accurate to avoid visible edges and other artefacts in the merged volume.

Furthermore we did not observe visible grey value variations in the merging area to compensate for. In addition the CT reconstruction parameters were kept identical to obtain consistent CT volumes.

In total the four CT scans of the airplane parts needed approximately 17 days to complete. To provide enough performance to permeate the airplane with X-rays, a 9 MeV linear accelerator X-ray source with a repetition rate of 400 Hz and a focal spot size of 2 mm was used. The distance between the X-ray source and the detector was set to *d*_S-D_ =  12 m and the source to rotational axis distance to about *d*_S-O_ = 10 m.

**Fig. 5:**
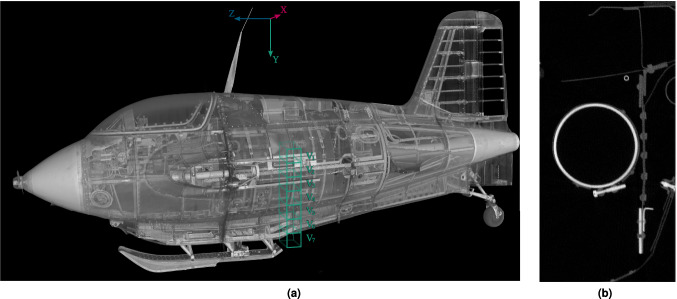
Rendering of the reconstructed fuselage of the scanned airplane (**a**) and detail (**b**) located at approximately the midpoint of the fuselage between the nose and the tail of the airplane. The green () boxes in **b** mark the positions of the extracted sub-volumes. The same Figure also shows the coordinate axis of the CT scan of the front part of the fuselage used for the sub-volumes.

The use of a line detector with a width of *w* = 4 m and a pixel spacing of 400 μm results in a horizontal resolution of 9,984 pixels. Using a vertical stepping motor, projective raw images with a spatial resolution of 9984 × 5286 pixels are obtained. The magnification of 1.2 leads to a horizontal voxel resolution of 330 × 330 μm^2^ and a vertical sampling of 600 μm within the reconstructed volume. Addressing the question of detail resolution and discernibility presents challenges, as these factors are influenced not only by voxel size but also by material contrast and data quality within the region of interest. As a heuristic approach, the minimum spatial extension of detectable object structures is generally on the scale of the voxel size. The obtained 16 bit data volumes of the two fuselage parts (see Fig. 5) have file sizes of $6,144\times 9,600\times 5,288$ voxels - or approximately 609 GB for the front part - and $6,144\times 9,600\times 5,186$ voxels - or 567 GB - for the rear part of the hull.

For tomographic reconstruction an adapted version of the Feldkamp-Davis-Kress (FDK) algorithm^14^ was employed. This modified FDK algorithm is tailored to the parallel fan-beam geometry of the XXL-CT system, with specific alterations made to the back projection and weighting functions to accommodate this distinctive setup.

As expected, and can be seen in Fig. 5b as well as the last column of Table 1, most of the airplane’s reconstructed interior consists of empty space or air. Apart from that, the CT volumes depict mainly a plethora of thin metal sheets, which have poorly or barely visible edge transitions to the adjacent metal sheets.

**Table 1:** Key metrics of the annotated sub-volumes depicted in Fig. 7.

Sub-volume	Coordinates in XXL-CT Volume	# Segments (prior post-processing)	Minimum segment size [voxel]	Maximum segment size [voxel]	Median segment size [voxel]	Foreground voxel [%]
*V*_1_	(3072, 4608, 0)	5 (5)	2,468	1,210,717	47,849	1.3
*V*_2_	(3072, 5120, 0)	7 (7)	1,277	1,173,579	**78,659**	1.1
*V*_3_	(3072, 5632, 0)	14 (13)	1,147	889,731	9,790	1.3
*V*_4_	(3072, 6144, 0)	33 (33)	1,293	**1,768,078**	2,217	4.5
*V*_5_	(3072, 6656, 0)	**169 (159)**	187	1,545,773	3,853	**9.4**
*V*_6_	(3072, 7168, 0)	108 (94)	**158**	1,567,273	4,039	5.6
*V*_7_	(3072, 7680, 0)	9 (9)	509	73,302	1,209	0.1

For the many cases where two metal sheets butt together, semantic information must be used to decide on the correct object boundaries between the entities. In addition, many regions in the XXL-CT volume are severely affected by artefacts from the data acquisition and reconstruction such as beam hardening or scattered radiation, especially in the vicinity of solid thick walled metal structures.

### Data annotation

Even though manual data labelling is currently referred to as the ‘gold standard’ for unique complex image data^15^, the required resources are quite high with respect to experienced staff and delineation time, even if specialized annotation pipelines (e.g.^16^ and ^17^) allowing image processing guided annotation, proofreading of inference results and model refinements, are applied for this task.

Hence, to reduce the costs of experts needed for manual or interactive image labelling tasks, so-known ‘crowd-sourcing’ approaches have been proposed and partially established^18–20^.

Nevertheless, to be effective, crowd-sourcing also profits strongly from specialized data management, annotation tool and soft skills of the annotators^21^. However, besides the huge amount of organizational, legal and logistic overhead, one drawback of crowd-sourcing is the limited understanding of the annotators about the annotation problem at hand and the complexity of the complex volumetric data depicting the various objects.

To somehow make a compromise between experts and crowd-sourcing, each individual sub-volume was initially annotated and labelled by an first annotator, and the thus acquired annotation was subsequently proofread and corrected by a second experienced annotator.

The complete annotation of the first two 512^3^ sub-volumes each needed about 350 working hours (or approximately two months with 40 hour per weeks), as the first annotator was trained on these sub-volumes and they contained many segments compared to later more empty sub-volumes. The manual annotation of each of these subsequent sub-volumes took about 10% to 50% of that time, mostly depending on the sub-volume complexity. The subsequent correction by different but trained annotators took about the same amount of time, or 4 to 120 hours per sub-volume.

The following Section Description of Data will give a brief overview of the annotated XXL-CT data, while the used annotation pipeline will be introduced in Section Annotation Pipeline.

#### Description of data

**Fig. 6:**
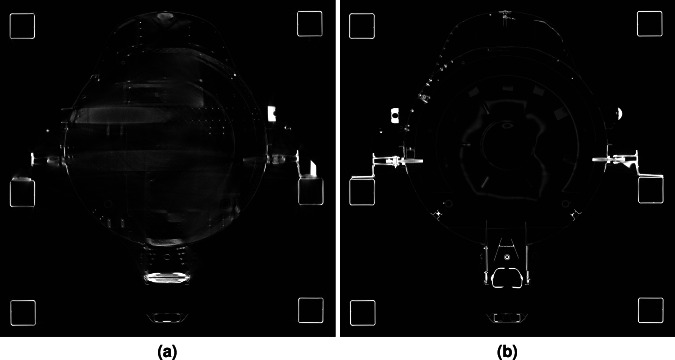
Slice of the airplane fuselage reconstruction volume near the rear of the pilot’s seat, where a bulkhead separates the cockpit from the rest of the fuselage. (**a**) displays significant imaging artefacts, while (**b**) presents an adjacent layer mostly free from such artefacts.

So far, seven 512 × 512 × 512 voxel sub-volumes of the Me 163 airplane have been annotated. The positions of these sub-volumes are indicated by the green boxes in Fig. 5a. The sub-volumes are positioned completely within the front section of the fuselage scan, touching but not crossing the boundary where the front and rear reconstruction volumes of the fuselage are joined together. The sub-volume blocks have been selected to cover an uninterrupted column from the underside of the fuselage almost to the top, without any overlap or gap between them. The selection of sub-volumes was made heuristically in a region that appeared representative of the data-set in terms of both the count and complexity of segments. The sub-volume positions were selected within regions of average data quality to mitigate the potential for artefacts (see Fig. 6). Given the inherent challenges associated with both manual annotation and automatic segmentation, this approach was deemed appropriate, as many regions of the airplane displayed comparable data quality. Artifacts in X-ray CT imaging are a complex and extensive field for research and development. They are caused by second order effects of the interaction of X-rays with condensed matter, e.g. by scattered radiation, spectral hardening of the X-ray beam, as well as inaccurate mechanical adjustments or geometrical approximations of the algorithms used for cross-sectional image reconstruction.

**Fig. 7:**
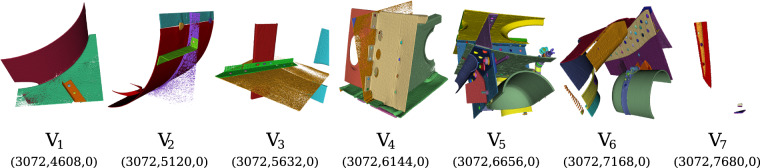
Examples of 3D-renderings of manually annotated and labelled sub-volumes from the XXL-Scan of the Me 163, depicting various semantic objects of different types, shapes, and materials.

Figure 7 provides examples of 3D-renderings of the seven annotated and labelled sub-volumes. Each sub-volume contains between 5 and 172 individual object entities of various sizes, materials, and types.

Table 1 gives a brief overview of the depicted objects in the annotated sub-volumes. Even though the regarded sub-volumes are located in the centre of the airplane (see Fig. 5a), the 0-coordinates in the second column indicate that they are placed exactly at the border between the two sub-scans. It can be seen, that the largest object in the Table 1, being a complex metal sheet, consisting of 1,768,078 voxels (with an equivalent of approximately 116 cm^3^), while the smallest object being a rivet contains only of 158 voxels (with an equivalent of 0.1 cm^3^). Both of these objects are bounded by their respective side surfaces of their surrounding sub-volumes and actually extend beyond them into adjacent sub-volumes. Overall annotated sub-volumes, approximately 93% of all voxels refer to background data, namely air, while only 7% (or 62.6 million voxels) relate to data of the depicted objects. These comprise a sum of 344 segments.

**Fig. 8:**
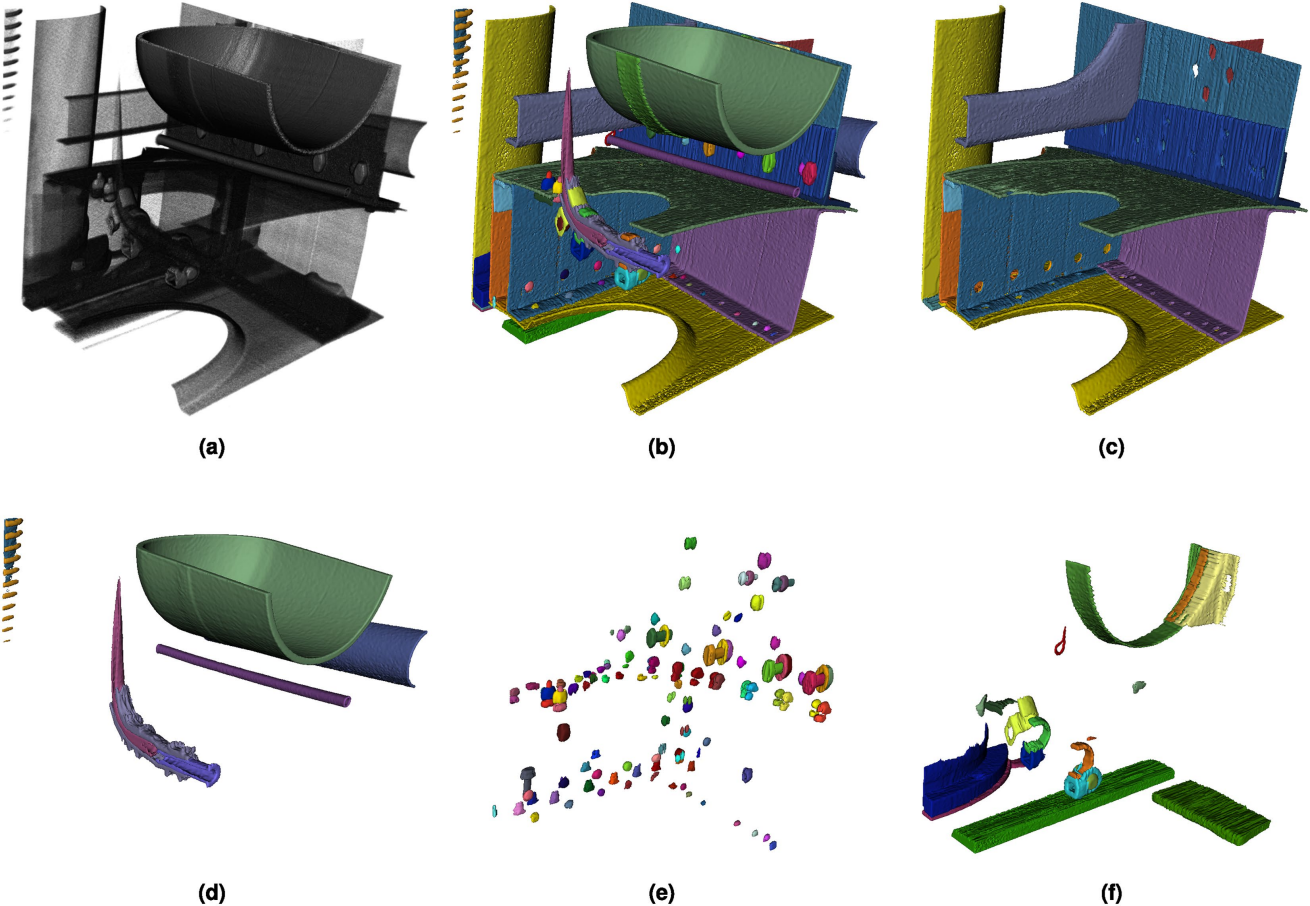
Example renderings of sub-volume *V*_6_ (3072,7168,0). While (**a**) shows the unannotated volume, (**b**) depicts all labelled segments separated by colour. To increase clarity, only the segments of a specific category are shown in the following sub-figures: (**c**) provides all metal sheets; (**d**) gives the presumably pressure-carrying pipes, pressure tanks and lines; (**e**) contains all rivets and screw connections; (**f**) finally shows all brackets, clamp connectors and other miscellaneous transition elements that could not otherwise be assigned a category.

Figure 8 shows different renderings of sub-volume *V*_6_ (3072,7168,0). While Fig. 8a depicts the unannotated volume, Fig. 8b shows all labelled segments separated by colour. To increase clarity, only the segments of a specific category are shown in the following: Fig. 8c provides all metal sheets; Fig. 8d gives the presumably pressure-carrying pipes, pressure tanks and lines; Fig. 8e contains all rivets and screw connections; Finally, Fig. 8f shows all brackets, clamp connectors and other miscellaneous transition elements that could not otherwise be assigned a category.

### Annotation pipeline

The annotation process of the XXL-CT data is on one side related to the used annotation software and annotation hardware. On the other side, it is highly dependent on the annotation rules and guidelines provided to the annotators as well as institutional knowledge which gets developed over time. Furthermore, some post-processing possibilities such as filtering, morphological operators, or data fusion must be considered.

#### Annotation software

We used the application *3D Slicer*^22,23^ for most of the annotation. This software provides the annotator with different types of interactive annotation tools such as paint strokes, boolean operations or grey value aware fill methods to select individual voxels and voxel groups. Furthermore, it can easily be extended with new segmentation functions^24^ and includes a powerful scripting interface.

#### Annotation hardware

**Fig. 9:**
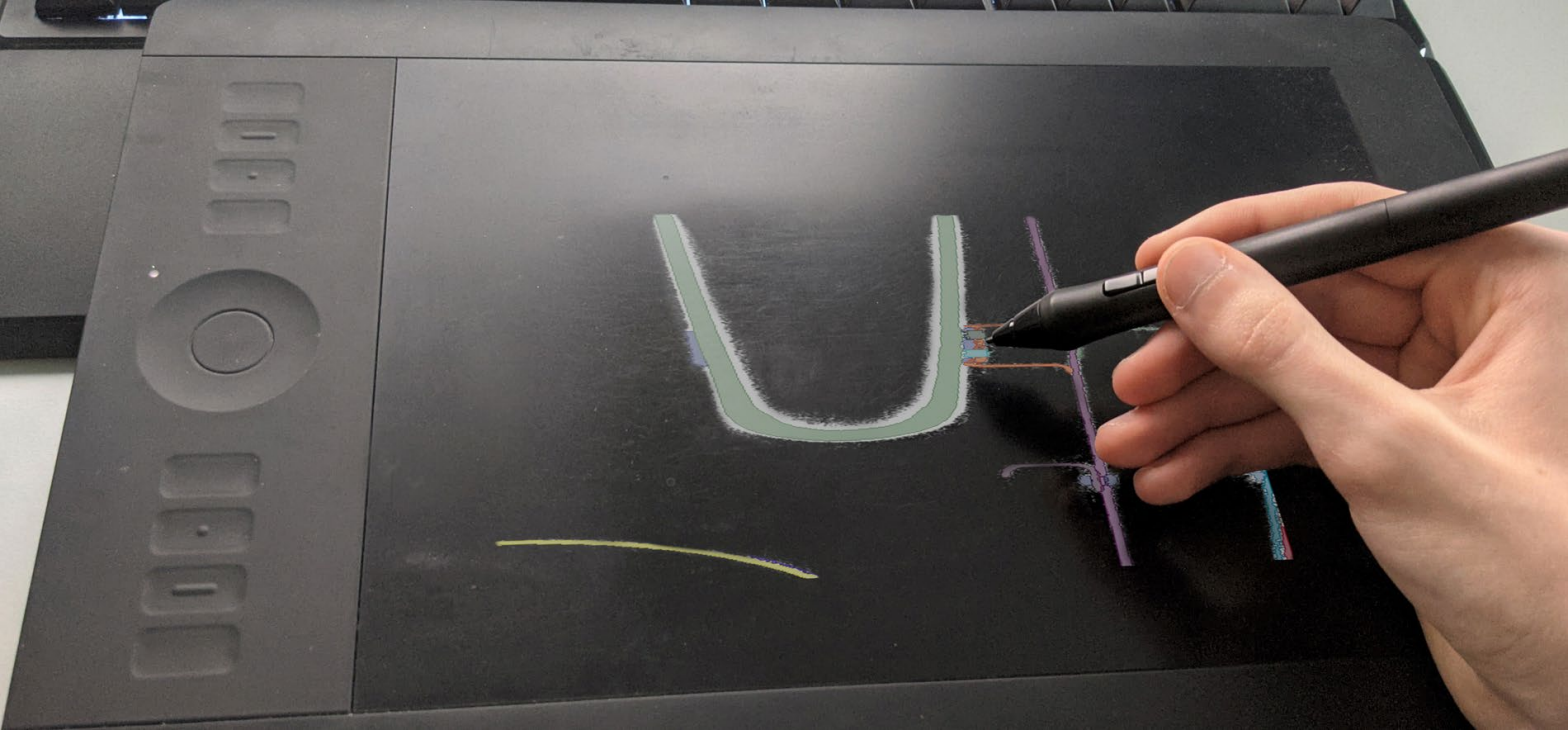
Manual labelling of large-scale industrial CT data of an airplane part using a high-resolution graphics tablet and a digital pen (Composed image to illustrate the process).

We used graphic tablets with digital styluses as input devices for the slice-by-slice manual annotation and labelling of the sub-volumes. As they allowed easy and intuitive drawing. In contrast to the use of a mouse this approach is more precise, intuitive and more importantly more gentle on the wrist of the annotators^25^. In Fig. 9 a typical manual segmentation and labelling task of a sub-volume from XXL-CT data can be seen using a graphics tablet.

#### Annotation guidlines

In our annotation guidelines, provided to all annotators, we stipulated that the ‘human interpreted reality’ of the data (based on the a-priori knowledge about the depicted objects) and not the ‘perceived visual representation’ should be segmented. For example, if scattered radiation artefacts were encountered, represented through bright or dark streaks through the volume or cupping artefacts from beam hardening, it was suggested to annotate the guessed *real specimen* and not the *distorted image*.

This should increase the uniformity of the annotations since otherwise it is difficult to find the same thresholds over different volume regions and artefacts. The ultimate goal of the work is to develop methods to separate all components from each other in a meaningful way. This may not be achievable in some cases, e.g. if there is not enough data available. However, this can only be known when everything has been tried, for which a meaningful annotation of the desired ideal result has to be available.

#### Annotation post-processing

After the individual segments have been partially annotated automatically by hand, they usually do not yet have the quality expected from a ground truth. Due to the presence of noise on the segment surfaces and voxels that were annotated as belonging to more than one segment, post-treatment is necessary.

##### Morphological closing

**Fig. 10:**
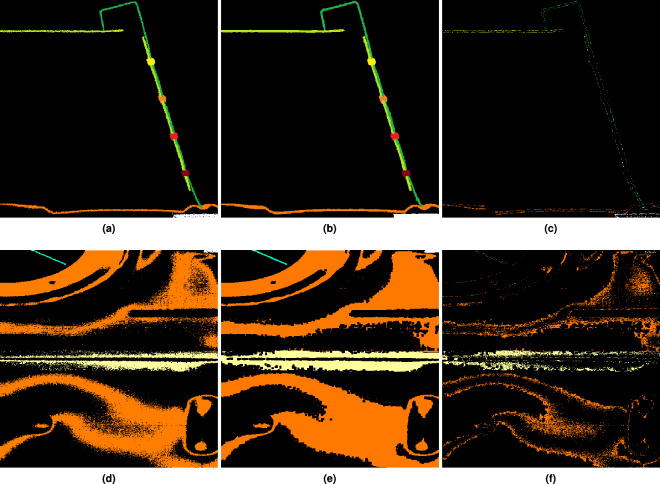
Slices from sub-volume *V*_4_ (3072,6144,0) depicting typical changes introduced by morphological post-processing. (**a** and **d**) (1st column) show manually annotated input volumes. (**b** and **c)** (2nd column) depict the morphologically postprocessed output. Finally, **c** and **f** (3rd column) show the difference between the input and output volumes. The upper row shows an example where the changes introduced by the post-processing consist mainly of small voxel alterations of the surface of a thin metal sheet. The bottom row depicts the changes close to the surface of the orange metal sheet located at the bottom of the upper row of images. This metal sheet appears to be quite noisy and therefore prone to the more pronounced changes visible in the residual Fig. 9f.

The use of the previously mentioned bandpass filter to visually smooth the grey values sometimes yields grainy textures inside the segments (see example in Section Noise). Due to the presence of this coarse-grained noise, we decided to postprocess the results obtained by manual annotation to close gaps between the quality of the manual annotation and the desired quality of the segmentation. Overall, it was aimed to achieve semantical reasonable and simultaneously visually pleasing segmentation results. For this purpose, the manual annotation of each segment was first postprocessed using a morphological closing filter^26^ with a 3 × 3 × 3 structure element. Figure 10 depicts two orthogonal slices from the manually segmented sub-volume *V*_4_ (3072,6144,0) prior and post morphological processing. While most of the changes introduced by the post-processing consist mostly out of simple surface voxel alterations (see Fig. 10c), they may also include changes to the surfaces of *noisy* metal sheets (see Fig. 10f) which are prone to the more pronounced changes due to their *noisy* nature.

##### Overlapping entities

We annotated each entity in the sub-volume individually slice by slice. In some rare cases, this yielded results, where we annotated voxels as belonging to multiple segments. For example, if the spatial resolution of the reconstructed volume data (with approximately 0.07 mm^3^ per voxel) was not sufficient enough to represent the exact border between two adjacent thin sheets of metal. It was not always possible to represent this cases in an annotation data-set with only voxel resolution. In such cases, the corresponding voxels were annotated as belonging to several segments.

After finishing the annotation and labelling process of all depicted entities in a sub-volume *V*_*i*_, all these segmented entities were combined on the voxel level into one single volume. Nevertheless, within this step we allowed the possibility to overwrite already existing voxels of previously included segments. The overwriting of labelled voxels primarily occurs at the edges between two adjacent segments. This means that the order in which the segments are processed and fused has partially influenced the result of the final segmentation results. Hence the order of the fusion sequence was assigned pseudo-randomly.

##### Connected component analysis

Finally, we performed a successive connected component analysis with a chessboard metric (aka Chebyshev distance or *L*_∞_ norm)^26^ to find the separated chunks. This also allows for a simple fix of the challenges described in Section Re-entering Segments. Furthermore, we discarded small segments with less than 100 voxels and deleted them, to avoid over-segmentation. The threshold of *θ* = 100 voxels was determined empirically.

### Challenges

**Fig. 11:**
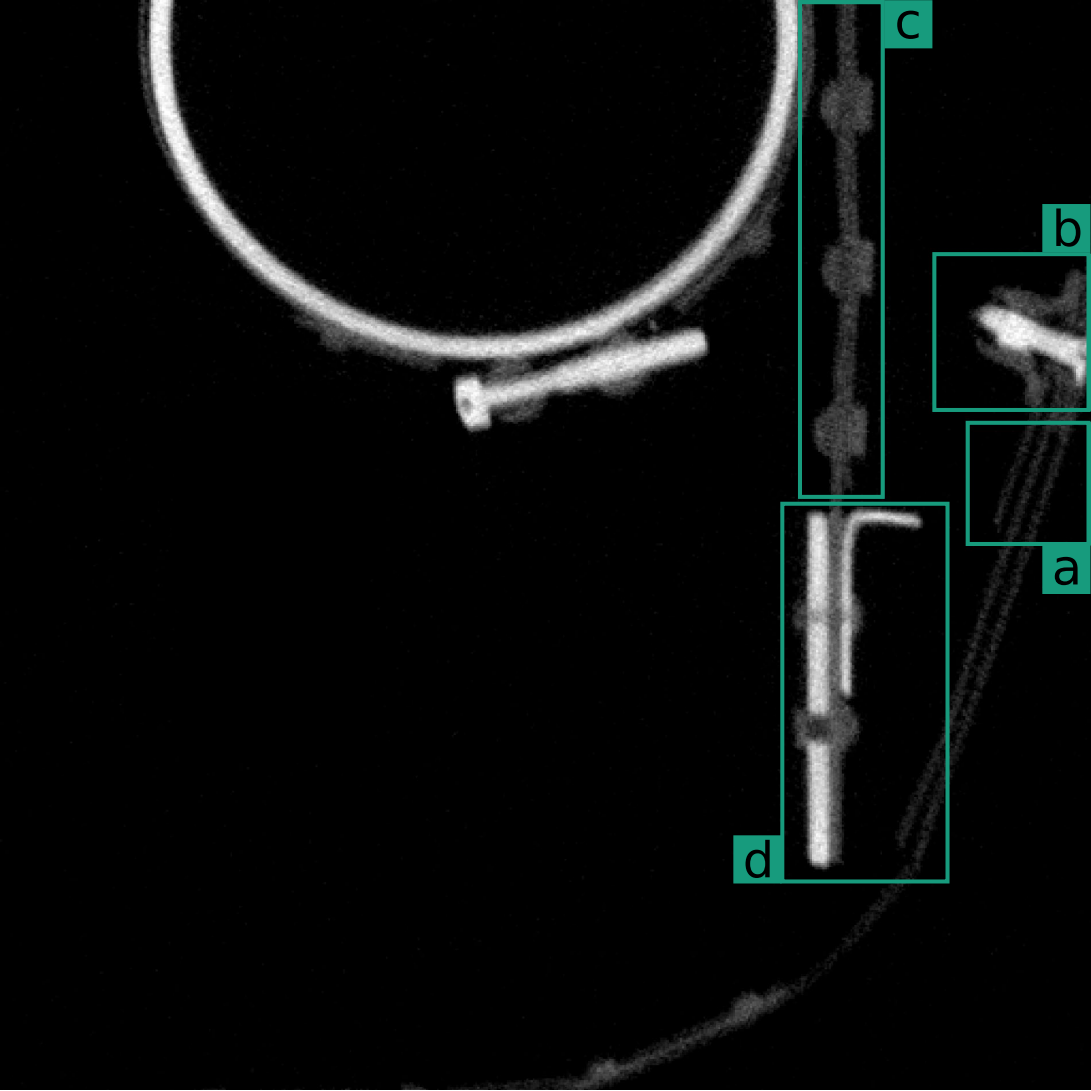
Sectional view from sub-volume *V*_6_ (3072,7168,0) in which some challenging areas have been marked: (**a**) noisy metal sheets (Section Noise); (**b**) low contrast (Section Low-Contrast Segment Boundaries); (**c**) low contrast and large contact area (also Section Low-Contrast Segment Boundaries); (**d**) annotator noise (Section Annotator Noise).

In the following section, we discuss, some characteristics of the above-introduced data-set and challenges regarding its annotation and labelling. Both, the XXL-CT imaging as well as the labelling steps provide ambiguities with respect to the data. To this end an example from sub-volume *V*_6_ (3072,7168,0) will be taken, see Fig. 11, and used as representative for the corresponding categories of challenges, namely Noise, low-contrast segment boundaries, segments leaving and re-entering the sub-volume as well as Annotator Noise. However, these categories are only exemplary and not to be understood as fully comprehensive.

#### Noise

**Fig. 12:**
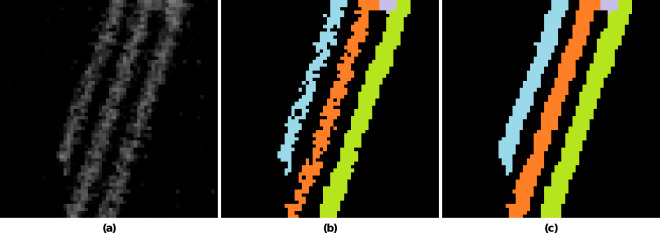
Three parallel metal plates with high noise in the reconstruction. (**a**) enlarged section from sub-volume *V*_6_ (3072,7168,0) (see Fig. 11(a)). The grainy texture is due to the low data quality and should therefore not be included in the annotation. (**b**) Result of naive segmentation; (**c**) desired segmentation after morphological closing.

Figure 11 shows at location (a) a region in which three parallel thin metal sheets are visible. In Fig. 12a an enlarged version is depicted where it can be observed that the three metal plates are interspersed with coarse-grained noise. Figure 12b provides the naive annotation strictly based on the visible grey values, leading to a result permeated by granular noise. However, using a-priori knowledge that the displayed metal components do not consist of sponge-like porous material, but the coarse-grained texture is due to measurement or reconstruction artefacts, the annotation is modified using the morphological closing (see above) as post-processing step, yielding the desired result shown in Fig. 12c.

#### Low-contrast segment boundaries

**Fig. 13:**
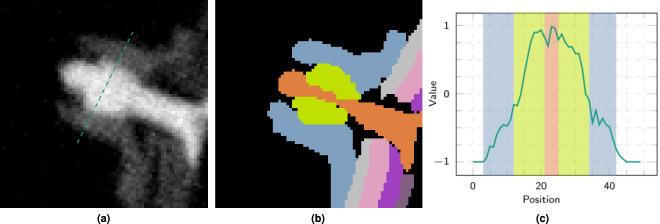
Example of low to no contrast entities. (**a**) Slice from sub-volume *V*_6_ (3072,7168,0) (see Fig. 11(b)) presumably showing a screw and its corresponding nut. No appreciable grey value and texture differences between the two components can be determined. (**b**) possible semantic annotation with an orange screw () and a light green nut () inside a blue structure (); (**c**) grey value profile plot along the green dashed section marked in the left subfigure, where the background colours indicate the possible annotation into the semantic segments.

Figure 11 at location (b) as well as the zoomed-in area in Fig. 13a shows a region in which specifically the bright object components to be annotated have no appreciable grey value or texture contrast to each other. Figure 13b shows a possible annotation in which the presumed bolt or screw (depicted in orange), runs through the nut (in light green). Figure 13c provides the grey value plot on along of the green dashed line in Fig. 13a. The coloured backgrounds refer to the annotation, see Fig. 13b. This annotation cannot be justified by the existing grey values and textures alone but must be made by examining the neighbouring similar structures and knowledge or assumptions about the production process.

**Fig. 14:**
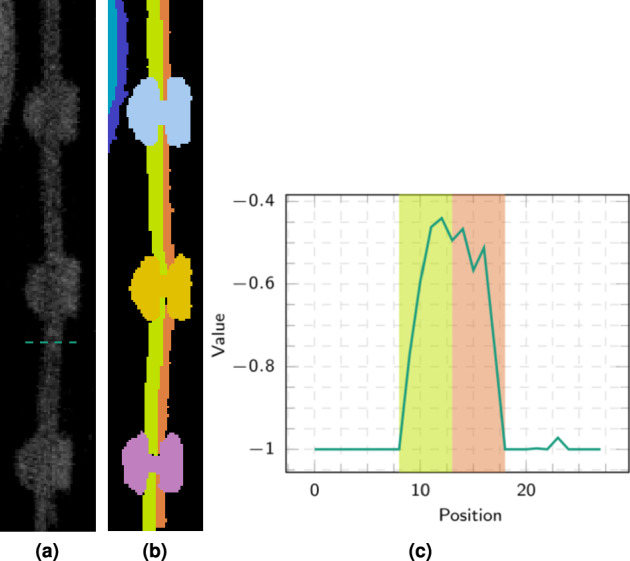
Example of low to no contrast entities. (**a**) Shows a slice from sub-volume *V*_6_ (2072,7168,0) (see Fig. 11(c)) containing two metal sheets riveted together. No appreciable grey value and texture differences between the two components can be determined. (**b**) Possible manual annotation of the left (light green ) and right (orange ) metal sheets. (**c**) Grey value plot along the green dashed section marked in (**a**). The background colors indicate the possible annotation into semantic segments.

Another example of such low-contrast segment boundaries between adjacent metal sheets is shown in the field of view in Fig. 11(c) or in Fig. 14. Figure 14b depicts a possible manual segmentation of the two metal sheets and the rivets in the regions. Figure 14c shows a grey value profile of the green dashed line shown in Fig. 14b, together with its possible segmentation as a colored background. Similar to before, the course of the segment boundaries can only be argued using a-priori knowledge from the surrounding segments and layers.

**Fig. 15:**
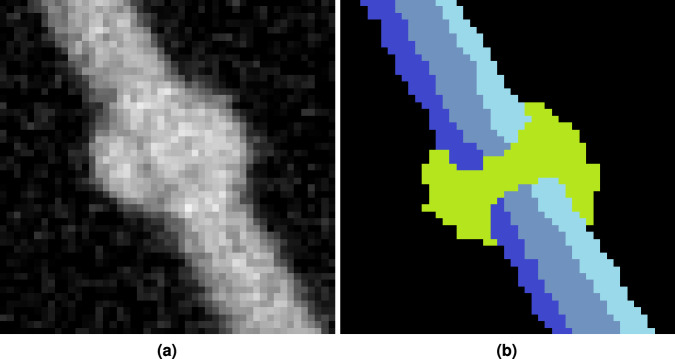
Example of an entity with low to no contrast. (**a**) slice from sub-volume *V*_3_ (3072,5632,0) presumably depicting three metal sheets riveted together. No appreciable visual grey values nor texture differences between the components can be determined. (**b**) possible (assumed) annotation of the regions taking the surrounding topology into account.

Finally, Fig. 15 shows a similar case from sub-volume *V*_3_. Here, a rivet penetrates three adjacent metal sheets. Due to similar material densities and the large and evenly shaped contact surface, the transition between the rivet and sheet metal cannot be discerned clearly.

#### Re-entering segments

**Fig. 16:**
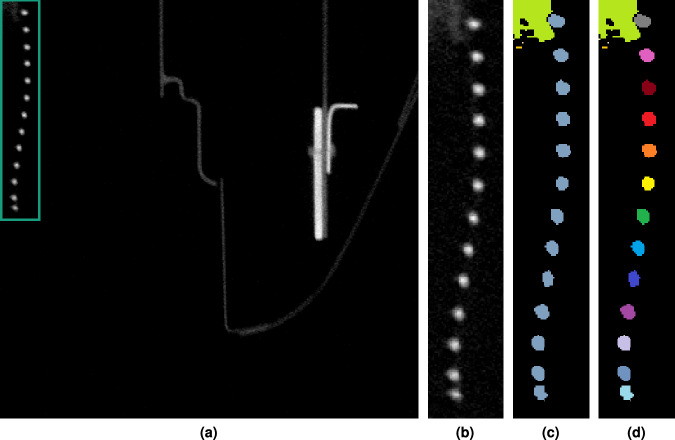
Example of a slice (from sub-volume *V*_6_ (3072,7168,0)) depicting a component which is not fully contained in the current sub-volume. (**a** and **b**) helical wire support structure. Without further information, the individual coils appear to be thirteen separate segments; (**c**) the result of human segmentation; (**d**) annotation result after connected component analysis, where no correspondences among the entities have been found.

Some components in the volumetric data leave the visible area of the current sub-volume and reappear as disconnected segments at a different location of the same sub-volume (see Fig. 16). Here the component of interest–a helical wire support structure probably for a suction hose–is located in the upper left corner of a sub-volume see Fig. 16a for overview and Fig. 16b for an enlarged view. Without any further semantic information, the individual coils appear to be thirteen separate segments. Figure 16c depicts the result of a human segmentation of these entities. Figure 16d provides the final annotation result after applying a connected component analysis, where no correspondences and connections among the thirteen entities have been found.

However, without additional semantic information about the course of the entities outside the sub-volume, it must be assumed that these segments are most likely separated from each other. For this reason, we performed a connected component analysis on the hand-annotated data-set and separated these segments as they leave and re-enter the sub-volume.

#### Annotator noise

Limited knowledge of the true real ground truth often leads to severe annotator noise^27,28^, which can often be observed within vast and difficult-to-label data-sets. Different annotators will have inconsistent knowledge of the problem domain, are possibly fatigued, subconsciously introduce their own bias into the annotation output, or will label multiple parts differently. Thus, the obtained annotation from a specific annotator or a fusion of several annotations should be only understood as one possible annotation.

**Fig. 17:**
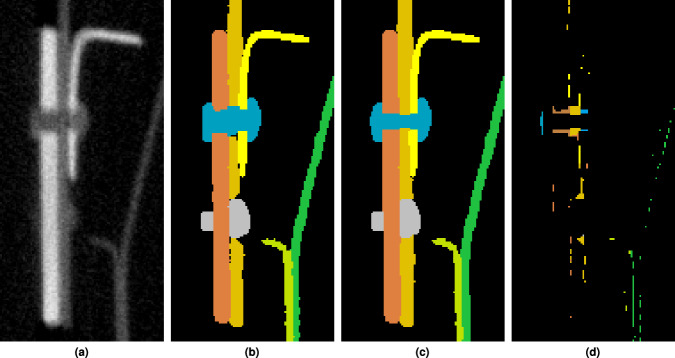
Example of a small region (from Fig. 11(d)) depicting multiple metal sheets riveted together annotated by two different annotators (**b** and **c**). The differences between both annotators are shown in (**d**).

Figure 17 shows a small region (Fig. 11(d)) of sub-volume *V*_6_ (3072,7168,0) which has been annotated by two different annotators (see Fig. 17b,c). The difference volume between the two annotations is depicted in Fig. 17d. As can be seen, most of the metal sheets only diverge in some surface voxels, whereas the rivet was annotated quite differently by the annotators.

## Data Records

The data-set comprises seven compressed archive files, which are accessible for download at 10.5281/zenodo.10651746^10^. Each archive is titled according to the respective sub-volume depicted in Fig. 7. The filename of each archive file includes both a consecutive sub-volume index (e.g., *V*_1,_ …,*V*_7_) as well as the Cartesian coordinates of the attachment point for the contained sub-volume within the coordinate system of the front fuselage reconstruction (e.g.,$(3072,4608,0),\ldots ,(3072,668,0)$). Each archive contains two subdirectories: *data* for the reconstruction and *target* for the annotation sub-volume. The sub-volumes are of the dimensions (512 × 512 × 512) voxels and are stored as losslessly compressed TIFF image files. Each image file represents an XY slice of the corresponding *data* or *target* sub-volume. The filename of each slice image indicates the offset Z-coordinate of the slice relative to the attachment point of the corresponding sub-volume. The reconstruction slice image files (*data*) consist of unsigned 16-bit integer greyscale voxel data. The annotation slice image files (*target*) comprise unsigned 32-bit integer voxel identifiers for individual segments. Within each sub-volume, these identifiers are uniquely associated with a single segment.

## Technical Validation

The example of a *segment correlation matrix* depicted in Fig. 19 shows how well the results of two different segmentations provided by two different annotators may match. In this case the *set of reference segments S*_*R*_ was initially generated by one annotator and is depicted on the vertical axis of the matrix in Fig. 19. The *set of detected segments S*_*D*_ was created by a second annotator, refining the first segmentation with our current understanding of the data-set. This set is depicted on the horizontal axis of the matrix in Fig. 19.

Each row is assigned to one *reference segment S*_*R*_ (*i*) and each column is assigned to a *detected segment S*_*D*_ (*j*). The value or colour of each cell corresponds to the *Intersection over Union* (*IoU*) score (also known as *Jaccard-Index*) of two segments *S*_*R*_ (*i*) and *S*_*D*_ (*j*):

$$IoU=\frac{| {S}_{R}(i)\bigcap {S}_{D}(j)| }{| {S}_{R}(i)\bigcup {S}_{D}(j)| }$$

If these two segments yield a complete overlap (meaning that their segmentations match completely) the value $IoU$ is equal to 1.0. If two compared segments do not share at least one common voxel, the value IoU will be 0.0. All other overlap scenarios are mapped to a value range of $IoU\in [0,1]$.

The rows in the matrix are sorted in descending order by the count of voxels of their corresponding reference segments. Consequently, the top rows correspond to the largest segments and the bottom rows to the smallest segments. The columns have been sorted by searching for the detected segment with the best match, or highest IoU to the reference segment of the current row. Each detected segment can only be assigned to a single reference segment. Detected segments unmatched to a reference segment are sorted by their voxel count. We excluded segments with a voxel count of fewer than 100 voxels to reduce the size of the matrix.

Hence, a *perfect segmentation S* with respect to a reference segmentation *R* should be reflected by a quadratic correlation matrix containing the same count of rows and columns, and thus the same amount of reference segments and detected segments. Additionally, all correlation values outside the main diagonal should contain IoU values of *IoU* = 0.0, while all values on the main diagonal should have values of *IoU* = 1.0. However, in realistic application examples, the row and column count will differ. Usually, an over-segmentation will result in more columns than rows. Boundary errors will result in suboptimal correlation values. Rows with multiple horizontal values either denote an over-segmentation of the respective detected segment, or a reference segment that was accidentally been split into multiple segments. In contrast, vertical lines indicate segments spanning multiple reference segments. They merge multiple reference segments. Breaks in the diagonal line indicate reference segments without a good match in the detected segments.

**Fig. 18:**
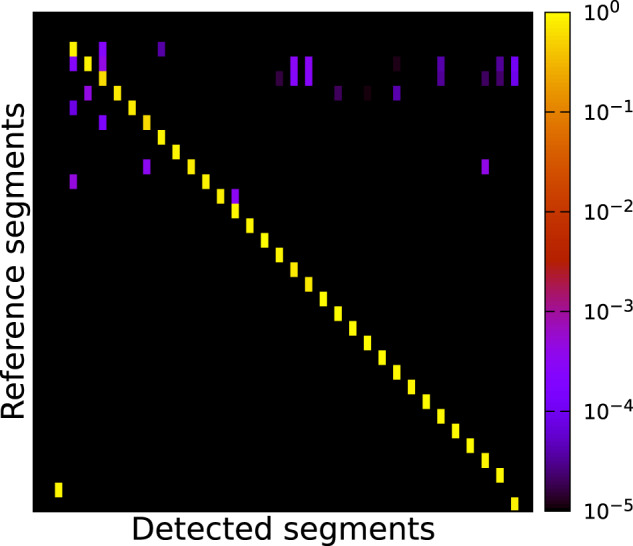
Correlation matrix of the segmentation of sub-volume *V*_4_ (3072,6144,0) before and after the post-processing. Rows correspond to *reference segments*, here the manual annotation (see Fig. 12b), which are sorted top to bottom by decreasing voxel count of the segments. The columns correspond to the *detected segments*, here the postprocessed segments (see Fig. 12c), which are sorted by the maximum IoU to a *reference segment*.

Figure 18 shows an example result of the manual annotation of a sub-volume compared to the postprocessed version of the same sub-volume. The desired bright diagonal line from the top left to the bottom right is pronounced, indicating that most of the *reference segments* (prior post-processing) could be assigned to the *detected segments* (after post-processing). The scattered purple cells, mostly located in the top third of the matrix, signal that some voxels of the manually augmented segments overlap multiple postprocessed segments and are assigned to them. This often happens if the surface of the manual segmentation which was created using the bandpass selection gets smoothed by the post-processing (Section Annotation Post-Processing).

**Fig. 19:**
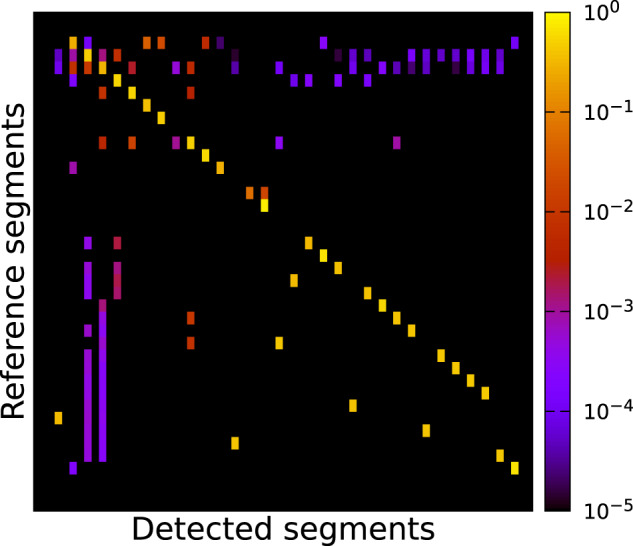
Correlation matrix of the segmentation of sub-volume *V*_4_ (3072,6144,0) of the segmentation results for the same data-set annotated by two different annotators. The rows correspond to *reference segments*, here the first initial annotation. The columns correspond to *detected segments*, here the second refined annotation, which are sorted by the maximum IoU to a *reference segment*.

Figure 19 shows the correlation matrix between the two manually annotated version of sub-volume *V*_4_ (3072,6144,0) which have been annotated by two different annotators. It can be seen that the annotated segments of the two annotators, especially the smaller segments, mostly match. As the two more or less pronounced vertical lines in towards the left matrix size indicate, most segments annotated by the first annotator lose voxels, most likely surface voxels, to the bigger segments segmented by the second annotator. The gap in the diagonal line almost at the center of the matrix corresponds to a rivet which was annotated much sturdier in the first annotation than by the second annotator.

## Usage Notes

In this work, we presented a data collection of seven manually-annotated sub-volumes obtained from an XXL-CT data-set from a historical airplane. These sub-volumes can potentially serve as a novel benchmark date-collection for instance segmentation in the field of non-destructive testing using XXL-CT sub-volumes. To our knowledge, at this point of time similar public data sets from XXL-CT are not available.

For the complete XXL-CT volume data we described the acquisition and measurement procedures, as well as its further processing. We described how and according to which criteria the seven sub-volumes were annotated and labelled manually by various annotators, including the description and discussion of challenges regarding possible ambiguities contained in the data-set.

We would like to note that although we have taken great care to annotate the sub-volumes to the best of our knowledge and belief, we may still have made mistakes. Some regions of the data-set simply cannot be clearly annotated due to the quality of the data and the recording modality.

All reconstruction and labelled sub-volumes are available under^10^.

We hope that the provided data sets are useful for further research.

## Data Availability

The data-sets generated during this work are available as multiple stacks of image files, which do not require any additional processing tools for use. The annotation of the data was carried out manually with semi-automatic post-processing, details of which are provided in the main text.
